# Awareness, perception and influences on uptake of the RTS,S/AS01 malaria vaccine among caregivers for children under 5 years in South West region, Cameroon

**DOI:** 10.5281/zenodo.18008087

**Published:** 2025-12-21

**Authors:** Jude Che Anye, Loveline Lum Niba, Omarine Njimanted, Eugene Enah Fang, Besong Tabot Itoe, Ebua Gallus Fung, Hermann Georges Ewi-Kang, Helen Kuokuo Kimbi

**Affiliations:** 1 Department of Public Health, University of Bamenda, Bamenda, Cameroon.; 2 Department of Microbiology and Parasitology, University of Bamenda, Bamenda, Cameroon.; 3 Department of Microbiology and Parasitology, University of Buea, Buea, Cameroon.; 4 Regional Technical Group for the fight against Malaria, South West Region of Cameroon.; 5 Department of Sociology and Anthropology, Faculty of Social and Management Sciences, University of Buea, Buea, Cameroon.; 6 Georgetown Global Health LLC., Yaounde, Cameroon.; 7 Department of Biomedical Sciences, University of Bamenda, Bamenda, Cameroon.

## Abstract

**Background:**

Malaria remains a major cause of morbidity and mortality among children under five in Cameroon. The recent introduction of the RTS,S/AS01 malaria vaccine provides an important opportunity to reduce the disease burden. However, little is known about caregivers’ awareness and perceptions during the early vaccine rollout. This study explored caregivers’ understanding, attitudes, and experiences regarding the malaria vaccine in selected urban and rural communities in the South West Region of Cameroon, forming part of a broader mixed-methods project aimed at informing strategies to strengthen vaccine uptake.

**Methods:**

An exploratory qualitative design was used, involving two focus group discussions with 20 mothers and caregivers of children aged 11–30 months. Participants were purposively selected to capture diverse perspectives across settings. Discussions examined awareness, perceived benefits, misconceptions, and contextual factors shaping vaccine uptake. Data were audio-recorded, transcribed, translated, and analysed thematically using an inductive deductive coding approach.

**Results:**

Findings showed that caregivers in the urban setting had high awareness of the malaria vaccine, though understanding was often limited and sometimes confused with other malaria interventions. Rural caregivers displayed uneven awareness but strong trust in health workers, which positively influenced acceptance. Across both settings, perceived benefits such as reduced severity of malaria episodes enhanced confidence in the vaccine. Barriers included communication gaps, misinformation, gender dynamics, long waiting times, and distance to health facilities. Caregivers recommended strengthening community-based communication through churches, town criers, health talks, outreach sessions, and visual materials.

**Conclusion:**

These findings highlight the importance of clear, consistent communication and trust-building as Cameroon expands malaria vaccine implementation. Enhanced community engagement and improved service delivery may support equitable uptake. The insights from this qualitative phase offer a foundation for further research within the wider mixed-methods project.

## Introduction

Malaria continues to be one of the most significant public health challenges in sub-Saharan Africa, disproportionately affecting children under five years of age. In 2023, more than 220 million malaria cases and over 600,000 malaria-related deaths were reported globally, with approximately 95% occurring in Africa and young children representing the majority of fatalities [1,2]. Cameroon remains among the highest-burden countries, with malaria constituting one of the leading causes of childhood morbidity and mortality and contributing to more than half of health facility consultations nationwide [2,3].

The introduction of the RTS,S/AS01 malaria vaccine represents a major milestone in malaria control. Evidence from Phase 3 trials demonstrated reductions in both uncomplicated and severe malaria among vaccinated children [4]. Early implementation data from pilot countries also showed promising uptake and reductions in severe disease [5]. Following WHO recommendations for malaria vaccine deployment, Cameroon integrated the RTS,S/AS01 vaccine into its Expanded Programme on Immunisation (EPI) in 2024, prioritising high-burden districts, including those in the South West Region where three out of 19 health districts were chosen [2,5].

Successful vaccine introduction, however, requires more than availability alone. WHO guidance emphasises the need for strong Social and Behaviour Change (SBC) approaches, effective communication, and community engagement to support informed caregiver decision-making and address misconceptions about novel vaccines [1]. Previous studies in sub-Saharan Africa show that vaccine uptake is influenced by caregiver knowledge, cultural beliefs, gender norms, trust in health workers, and health system factors such as accessibility and service quality [6-8]. Understanding these contextual factors is essential, especially during the early phases of vaccine rollout.

In addition, WHO and global immunisation partners recommend the use of Behavioural and Social Drivers (BeSD) frameworks and Social & Behaviour Change (SBC) approaches when introducing new vaccines. These frameworks stress rapid formative assessment, clear and repeated messaging that distinguishes the new vaccine from existing malaria interventions, use of trusted local communicators, and monitoring of demand‑side drivers measures that directly address the types of awareness and confusion observed in this study [9].

WHO’s malaria vaccine policy and the accumulated evidence from the Malaria Vaccine Implementation Programme (MVIP) emphasise that introduction of RTS,S/AS01 must be accompanied by robust programme planning, monitoring, and community engagement to realise public health benefits. The WHO position paper and the SAGE/ MPAG evidence reviews summarise both vaccine impact from trials and operational lessons from pilot introductions in Ghana, Kenya and Malawi, where routine delivery reached millions of children and generated important implementation learning [10].

Despite the potential benefits of the malaria vaccine, there is limited evidence on how caregivers in Cameroon understand and perceive it within their local contexts. Differences between urban and rural communities, varying access to information, and long-standing beliefs about malaria prevention can shape acceptance and uptake. Early insights from Cameroon’s pilot rollout suggest that caregivers’ awareness and trust in health workers may play a significant role in vaccine adoption [11].

This study forms part of a broader mixed-methods project aimed at strengthening malaria vaccine uptake among children under five in the South West Region of Cameroon. The qualitative component presented here explores caregivers’ awareness, perceptions, perceived efficacy, and contextual influences on uptake of the malaria vaccine. As an exploratory inquiry, the findings are not intended to provide conclusive determinants but rather highlight key issues requiring further investigation and identify areas that can guide the development of context-appropriate strategies for promoting malaria vaccine uptake.

## Methodology

### Study design

This study employed an exploratory qualitative design to examine caregivers’ awareness, perceptions, and experiences related to the malaria vaccine.

Data were collected in two purposively selected communities, one urban and one rural, within high-burden health districts participating in Cameroon’s pilot introduction of the RTS,S/AS01 malaria vaccine [5]. The selection of contrasting settings enabled examination of contextual differences that could influence awareness and uptake.

### Study population and sampling

The study was a community-based qualitative study with the study population made up of mothers/caregivers of children 0-5 years residing in the selected communities. Purposive sampling was used to recruit participants with diverse demographic characteristics to enrich the range of perspectives. A total of 20 caregivers participated in two focus group discussions (FGDs); one urban and one rural each comprising 10 participants. Although qualitative studies do not rely on statistical representativeness, two FGDs were considered adequate for the few health districts piloting the vaccine within the region. This was due to thematic sufficiency, resource constraints, and the aim to capture contrasting community experiences.

### Inclusion and exclusion criteria

Included were mothers/caregivers of children 11-30 months of age at the time of data collection. Excluded were mothers/caregivers with no information on their child’s vaccination status, who did not provide their consent to participate in the study.

### Sample size determination

Purposeful technique was used to get participants within the two FDGs. Participants were identified through collaboration with community health workers and local leaders. Health workers collaborated with community leaders to help identify and mobilise participants from the different rural and urban settings coming from diverse backgrounds and communities within the different localities.

### Data collection instruments

An FGD guide was designed pertaining to different themes and used at the points of data collection to get respondents' perceptions on malaria vaccine awareness, acceptance, and uptake as well as their associated factors in other to propose recommendations that can be implemented to guide health care providers on the optimisation of the malaria vaccine uptake. This FGD guide was adapted from previous qualitative studies like the current research with some modifications done to suit the context of the study [12-14]. FGDs were conducted in English and Pidgin English depending on participant preference. Each lasted 90–100 minutes, was audio recorded with permission, and complemented by field notes.

### Pre-testing of tool

The data collection tool designed by the investigator was pre-tested two weeks before the start of the main research amongst six caregivers in Bonjongo health area in the south west region which wasn’t part of the study to ensure clarity and cultural appropriateness. Minor adjustments were made following feedback.

### Transcription and translation

Recordings were transcribed verbatim. Pidgin English responses were translated into English by bilingual researchers. Accuracy was ensured through cross-checking by an independent reviewer.

### Data management and analysis

Thematic analysis following Braun and Clarke’s phases was used. An inductive–deductive approach guided coding. A preliminary codebook was developed and applied using NVivo 12. Two analysts independently coded transcripts, with 10% double-coded for triangulation. Themes were refined collaboratively. This was to reduce bias and revise the themes that might have occurred due to discrepancies and unexpected findings. The entire team subsequently reviewed the generated themes to ensure that they reflect respondents’ ideas as opposed to the likelihood of bias often associated with a single analyst.

### Ethical considerations

Ethical clearance was be obtained from the FHS Institutional Review Board (Ref. 2025/0002H/ Uba/IRB) and the Regional Ethics committee for Human Health research in the South-West region of Cameroon (Ref. No771/CRERSH/SW/C/09/2025. A signed authorization to carry out research was obtained from the South West regional Delegation of Public health, Cameroon. A written consent form was signed by each participant before the start of the FDG. The goal of the study and procedure was explained to each participant of the study. After the signing of consent form by a participant, a verbal consent was obtained by the investigator from participants for recording of information given through an audio recorder. Confidentiality was maintained using ID codes. Names of participants and the facilities where they work were not disclosed. Participants were also given the free will to express themselves in any language of their choice. There was no major risk in participating in this study. The purpose of the study and procedures were explained to all participants in their local language at the time of recruitment.

## Results

### Socio-demographic characteristics of participants

The 20 participants that took part in the 2 FGD from rural and urban settings were residents of different quarters within the health areas concerned. Their ages ranged from 20 to 53 yrs, with the majority being young adults between 20 and 34 years old. All participants were female caregivers of children <5 years, with most caring for one child aged between 11 months and two years. One participant cared for twins, and another (aged 53) cared for a child who was not her biological child. Educational levels varied, ranging from primary education to high school, with several participants having completed secondary education, and three having attended vocational institutions. Most of the participants were self-employed and engaged in diverse occupations including farming, seamstress work, hairdressing, decoration, and various business activities. Three participants reported having no current employment.

### Urban setting

Caregivers in the urban community demonstrated high awareness of the malaria vaccine; however, this awareness was often superficial. Participants frequently confused the malaria vaccine with other malaria-related interventions such as IPTp or malaria treatment drugs. This reflects inconsistencies in health education and message clarity at health facilities (Table 1).

**Table 1:** Brief overview of malaria vaccine awareness/knowledge in the urban setting.

Areas of focus	Findings	Interpretation
Awareness of malaria vaccine	All mothers had heard of the malaria vaccine	High level of awareness
Correct understanding of what the vaccine is	Low	Participants confuse vaccine with preventive / treatment drugs
Knowledge on who receives the vaccine	Mixed	Some said mothers received the vaccine instead of infants protecting them and their babies
Knowledge of vaccine schedule	Inaccurate and inconsistent	Indicate limited vaccine literacy
Source of information	Many knew from the health facilities	Reliance on health facility level communication structures

Fear and misconceptions were common, including associations with COVID-19 vaccines and beliefs about population control. Household gender dynamics also influenced decision-making, as some caregivers required approval from their husbands before accepting vaccination.

Despite misconceptions, caregivers acknowledged the perceived benefits of the vaccine, such as reduced severity and frequency of malaria episodes. Nevertheless, structural barriers including distance to facilities, long waiting times, and negative staff attitudes impeded optimal uptake. Trust in the health system varied. Caregivers trusted known health workers and visual communication tools but expressed skepticism about perceived favouritism and inconsistent staff presence.

Participants recommended stronger community-level sensitisation through churches, posters, social media, and outreach efforts targeting hard-to-reach areas (Table 2).

**Table 2:** Thematic analysis and quotes from the FGD in an urban setting.

Themes	Sub-themes	Direct quotes	Interpretation
Awareness and knowledge of the malaria vaccine	Conceptual misunderstanding	*“I think many of us here are misunderstanding this malaria vaccine with other preventive measures. Some of us are describing treatment measures our children received.” (P8)*	Participants are aware of the term “malaria vaccine” but confuse it with other preventive or treatment interventions, showing partial understanding rather than true awareness.
	Inconsistent health education	*“We have not even heard much about the vaccine because sometimes when we come to the clinic, they don't give health talks and some other times they give health talks when some of us might not be there.” (P1)*	Health education and sensitisation on the malaria vaccine are inconsistent, leading to uneven community awareness. This reflects structural gaps in information delivery.
Fear and misconceptions about the vaccine	Association with COVID-19 and population control	*“Some of us were afraid and missed doses because we thought it was that corona virus which they wanted to inject into us.” / “We were afraid because we thought they wanted to reduce population through the vaccine.” (P6)*	Misinformation and conspiracy beliefs (linking malaria vaccine to COVID-19 or population control) drive fear and hesitancy
	Patriarchal influence on decision-making	*“We cannot take it if our husbands have not approved where it's coming from at the community.” (P3)*	Male authority and household decision hierarchies significantly influence women’s acceptance of vaccination.
Perceived importance and efficacy	Observed reduction in malaria episodes	*“Since after the vaccine, my child has been having fever sometimes but when they test at the hospital, they say the child does not have malaria which was not the same before.”(P7)*	Positive lived experiences reinforce trust in the vaccine’s efficacy, acting as facilitators of uptake.
Barriers to vaccine uptake	Distance and transport difficulties	*“Some of us are living very far and we find difficult getting to facilities.” (P7)*	Physical distance limits access to vaccination services, in urban areas.
	Health worker attitude	*“Sometimes they speak to us harshly and tell us to come another day and it discourages us.”(P2)*	Negative staff attitudes discourage return visits and reduce vaccine adherence.
	Service load and inequality	*“Few staff led to long waiting times and rescheduling appointments.” / “Perceived unfairness in distribution of mosquito nets and other services.”*	Systemic inefficiencies and perceived favoritism reduce community trust and engagement.
Mixed trust in health facilities and personnel	Selective trust based on fairness and familiarity	*“I trust them but there are certain things that I don't trust. They give more mosquito nets to their friends and families.”* *“Fear comes up when they change the nurses and new faces appear.” (P7)*	Trust is conditional; perceived corruption or bias undermines confidence in healthcare systems.
	Trust linked to good communication	*“I trust them because when they explain with posters and images, I know it is legitimate.” (P4)*	Clear visual communication and transparency help to build confidence in health services.
Community awareness campaigns needed	Multi-channel outreach	*“They should send it to social media because youths today are versed with Facebook and Tiktok.” (Group)*	Participants desire modern and accessible outreach methods beyond traditional health talks.
	Rural inclusion	*“They should go to inside those ‘Banga bush’ places and not only town roads.” (P1)*	Awareness campaigns must reach marginalised rural communities to ensure equitable access to information.

### Rural setting

In rural communities, awareness of the malaria vaccine was uneven. Some caregivers learned about the vaccine through health centers or town criers, while others lacked prior information. Despite this, trust in nurses and midwives remained exceptionally strong and was a critical facilitator of acceptance. Caregivers consistently perceived the vaccine as beneficial, noting that vaccinated children experienced milder malaria episodes and faster recovery. Barriers to awareness were primarily informational: caregivers often missed announcements due to farming schedules or could not hear town crier messages clearly. Participants suggested strengthening communication through churches, improved outreach messages, and clearer identification of which vaccines were being administered during campaigns (Table 3).

**Table 3:** Thematic analysis and quotes from the FGD in a rural setting.

Theme	Sub-themes	Direct quotes	Interpretation
Awareness	Health center as main source of information	*“I heard about it here at the Health Center.” (P1)*	Health centers are trusted and primary points for vaccine information.
	Community announcements	*“The town crier goes around to tell people when the vaccination will take place.” (Group)*	Town crier announcements are key but inconsistent in reach.
	Missed announcements	*“Maybe when they announced it, I was not around, so I didn’t hear.” (P4)*	Missed communication due to absence or limited reach.
Perception	Perceived effectiveness	*“When malaria affects a child who has taken the vaccine, the illness is not as serious.” (P12)*	Mothers perceive reduced severity of malaria after vaccination.
	Observational evidence	*“A child who has received the vaccine and one who has not are very different.” (P12)*	Belief strengthened through observation and peer experience.
Trust	Trust in nurse/ midwife	*“The person who told me about the vaccine is someone I trust Mummy Sophia.” (P7)*	Trust in local healthcare personnel drives vaccine acceptance.
	Trust in the health facility	*“Anything coming from the the Health Center is something good.” (P12)*	Institutional trust contributes to high perceived safety.
Fear	Lack of fear	*“No, we are not scared of the vaccine.” (P4)*	Fear not a significant barrier; caregivers show openness.
Communication gaps	Confusion about vaccine type	*“They just said, ‘Come for vaccination,’ without telling us which type.”*	Need for clearer vaccine-specific communication.
Barriers	Missed Outreach	*“Sometimes the town crier just passes on the road, but we don’t always hear what he says.” (P3)*	Ineffective communication reach to some households.
Suggestions	Church announcements	*“They should also make announcements in churches.” (P3)*	Churches identified as effective alternative communication channels.
	Clear vaccine identification	*“They should make clear announcements… so mothers can know which specific vaccine is being given.” (P3)*	Improves vaccine literacy and transparency.

## Discussion

This exploratory qualitative study provides insight into caregivers’ awareness, perceptions, and experiences related to the malaria vaccine within selected urban and rural communities of the South West Region of Cameroon. The findings highlight both shared and context-specific influences on vaccine uptake during the early phase of Cameroon’s malaria vaccine rollout.

Overall, caregivers expressed willingness to vaccinate their children; however, understanding of the malaria vaccine varied across settings. Urban caregivers displayed high but often superficial awareness, frequently confusing the vaccine with other malaria interventions. This reflects broader findings that overlapping health messages can make it challenging for caregivers to differentiate between preventive strategies [6,7].

In rural settings, awareness was uneven but acceptance was shaped strongly by trust in some nurses, midwives, such as “Mummy Sophia”, who are seen as community protectors and whose guidance is followed without fear or doubt. This deep trust has minimised vaccine hesitancy, a common challenge elsewhere. This aligns with evidence showing that interpersonal communication from known and trusted health workers is central to vaccine acceptance in low-resource settings [7,8,15]. Trust functioned as both a motivator and a buffer against knowledge gaps.

Across both settings, perceived benefits such as reduced malaria episodes and faster recovery reinforced positive attitudes towards the vaccine. These observations echo findings from clinical and implementation studies that show a reduced malaria burden among vaccinated children [4,5] and support behavioural theories linking perceived benefit to proactive health-seeking behaviour [16].

Multiple barriers influenced uptake, including long distances to facilities, competing responsibilities, long waiting times, and perceived negative attitudes from health workers. Similar systemic challenges have been identified in other African settings, highlighting the need to strengthen service delivery and communication to support malaria vaccine uptake [6,7].

Fear and misinformation emerged as key drivers of vaccine hesitancy. Misconceptions linking the malaria vaccine to the COVID-19 vaccine or population control reflect the lasting effects of pandemic-related mistrust. Cultural and gendered decision-making norms also play a significant role as some women deferred to male household heads before accepting vaccination. These findings highlight the influence of social structures and the need for male engagement and community leaders in vaccine advocacy. These findings are corroborated by Jenna *et al.* [17] and Nyalundja *et al.* [18] who also found that these factors significantly influenced vaccine acceptability.

Physical and systemic barriers including long distances, poor transport, overburdened health facilities, and negative staff attitudes hamper vaccine uptake. The combination of structural challenges and interpersonal discouragement reveals the importance of health system strengthening and staff capacity building. Similar barriers were identified by Simbeye *et al.* [19] in Malawi, who found that longer travel distance and prior adverse reactions reduced uptake of the malaria vaccine.

The findings suggest the need for strengthened, clear, and consistent communication using multiple channels like social media, churches, community gatherings, posters, and health talks to ensure caregivers receive accurate and timely information. WHO guidance emphasises integrated Social and Behaviour Change (SBC) approaches, which could support clarity and improve vaccine literacy [1].

Leveraging trust in frontline health personnel is essential, particularly in rural areas. Ensuring continuity of staff, respectful care, and adequate time for counseling could foster further confidence in the malaria vaccine [9].

Long waiting times and distance to facilities highlight the need for improved clinic workflow, equitable resource distribution, and enhanced outreach services to reduce missed vaccination opportunities [10,17].

### Limitations of the study

This study had several limitations. First, it was based on only two FGDs (one urban and one rural), which may limit the breadth of perspectives captured. Nevertheless, the discussions yielded recurring themes that provide important preliminary insights. Second, the study focused exclusively on caregivers’ perspectives. Healthcare workers’ and managers’ perspectives, though part of the broader mixed-methods project, were not included here and will be reported separately. Despite these limitations, the findings offer valuable early evidence to inform communication, service delivery, and community engagement strategies as Cameroon prepares to expand malaria vaccine implementation.

## Conclusions

Perceived benefits, including reduced malaria severity and faster recovery among vaccinated children, contributed to positive attitudes, while communication gaps and structural challenges such as long waiting times and distance to health facilities influenced uptake. As part of a broader mixed-methods project, these qualitative findings provide foundational in-sights that can inform future investigation and guide context-appropriate strategies aimed at strengthening malaria vaccine uptake and ensuring equitable access.

### Recommendations

- Enhance clarity and consistency of communication by strengthening health education during antenatal care, child welfare clinics, and vaccination sessions to differentiate the malaria vaccine from other malaria interventions.

- Expand community-level communication channels, including churches, women’s groups, community outreaches, town criers, and digital platforms, to improve the reach and accuracy of vaccine information.

- Leverage trusted health personnel such as nurses, midwives, and community health workers to deliver vaccine messaging and address caregivers' concerns, especially in rural areas.

- Strengthen interpersonal communication and respectful care within health facilities to enhance caregiver trust and reduce hesitancy.

- Address structural and service delivery barriers by improving clinic workflow, reducing waiting times, and enhancing outreach services for hard-to-reach areas.

- Prioritise continuous research and monitoring to further understand barriers and facilitators of uptake, strengthen implementation strategies, and evaluate progress during scale-up.
